# Quantitative Structure-Activity Relationships Study on the Rate Constants of Polychlorinated Dibenzo-*p*-Dioxins with OH Radical

**DOI:** 10.3390/ijms160818812

**Published:** 2015-08-12

**Authors:** Chuansong Qi, Chenxi Zhang, Xiaomin Sun

**Affiliations:** 1 College of Chemical Engineering, Beijing Institute of Petrochemical Technology, Beijing 102617, China; E-Mail: qichuansong@bipt.edu.cn; 2 Environment Research Institute, Shandong University, Jinan 250100, China; E-Mail: sdzhangcx@163.com; 3 Department of Resources and Environment, Binzhou University, Binzhou 256600, China

**Keywords:** polychlorinated dibenzo-*p*-dioxins, rate constants, number and position of Cl atom, quantitative structure-activity relationships

## Abstract

The OH-initiated reaction rate constants (*k*_OH_) are of great importance to measure atmospheric behaviors of polychlorinated dibenzo-*p*-dioxins (PCDDs) in the environment. The rate constants of 75 PCDDs with the OH radical at 298.15 K have been calculated using high level molecular orbital theory, and the rate constants (*k*_α_, *k*_β_, *k*_γ_ and *k*_OH_) were further analyzed by the quantitative structure-activity relationships (QSAR) study. According to the QSAR models, the relations between rate constants and the numbers and positions of Cl atoms, the energy of the highest occupied molecular orbital (*E*_HOMO_), the energy of the lowest unoccupied molecular orbital (*E*_LUMO_), the difference Δ*E*_HOMO-LUMO_ between *E*_HOMO_ and *E*_LUMO_, and the dipole of oxidizing agents (D) were discussed. It was found that *E*_HOMO_ is the main factor in the *k*_OH_. The number of Cl atoms is more effective than the number of relative position of these Cl atoms in the *k*_OH_. The *k*_OH_ decreases with the increase of the substitute number of Cl atoms.

## 1. Introduction

Polychlorinated dibenzo-*p*-dioxins and polychlorinated dibenzofurans (PCDD/Fs) in the atmosphere mainly derive from the processes of waste combustion, including carbon source and chlorine [1,2,3,4]. Due to the fact that PCDD/Fs tend to bioaccumulate and biomagnify in the food chain, PCDD/Fs have been a frequent topic in the public discourse [5,6]. The OH radical reactions with PCDD/Fs are considered a dominant removal process in the atmosphere [7,8].

The OH radical reaction rate constants (*k*_OH_) play a significant role in measuring the atmospheric behaviors of organic pollutants in the environment, and are indispensable for environmental risk assessment of organic chemicals. The *k*_OH_ can be used to calculate the half lives of most organic compounds in the air. Thus, it is critical to know the *k*_OH_. Unfortunately, due to the high toxicities and rather low vapor pressure of PCDD/Fs, direct kinetic measurements of the reactions of PCDD/Fs and OH radical are scarce. Only a few experimental reports are available for the gas-phase reactions between PCDD/Fs, for example, Brubaker and Hites [9] measured the *k*_OH_ of dibenzo-*p*-dioxin (DD), 2,7-dichlorodibenzo-*p*-dioxin (2,7-DCDD), dibenzofuran (DF) and 2,8-dichlorodibenzofuran (2,8-DCDF) in a small, heated quartz reaction chamber sampled by on-line mass spectrometry. Taylor *et al*. [10] obtained the *k*_OH_ of DD, 2-chlorodibenzo-*p*-dioxin (2-CDD), 2,3-dichlorodibenzo-*p*-dioxin (2,3-DCDD), 2,7-DCDD, 2,8-dichlorodibenzo-*p*-dioxin (2,8-DCDD), 1,2,3,4-tetrachlorodibenzo-*p*-dioxin (1,2,3,4-TCDD) and octachlorodibenzo-*p*-dioxin (OCDD) using the pulsed laser photolysis/pulsed laser-induced fluorescence (PLP/PLIF) technique. In recent years, computational chemistry has emerged as a potent tool to identify rate constants. Altarawneh *et al*. [11] obtained a rate constant of 2.70 × 10^−11^ cm^3^·molecule^−1^·s^−1^ for DF with OH radicals using conventional transition state theory (TST). Wang and Tang [12] have estimated the *k*_OH_ of DD, 1,4-dichlorodibenzo-*p*-dioxin (1,4-DCDD), 1,6-dichlorodibenzo-*p*-dioxin (1,6-DCDD), 1,9-DCDD, 2,3-DCDD, 2,7-DCDD, 2,8-DCDD, 1,4,6,9-tetrachlorodibenzo-*p*-dioxin (1,4,6,9-TCDD), 2,3,7,8-tetrachlorinated dibenzo-*p*-dioxin (2,3,7,8-TCDD) and OCDD with the same method, which showed that the *k*_OH_ of these PCDD/Fs were found closely related to the number and position of chlorine (Cl).

Therefore, this study was aimed to obtain the *k*_OH_ database of PCDDs at 298 K. Based on our findings, we employed the quantitative structure-activity relationships (QSAR) model to establish the relation between the *k*_OH_ and the molecular structure of the PCDDs. The technology of QSAR have been developed to establish relationships between the properties and the molecular structure of the compounds [13,14,15] and predict the rate constants for the reactions of organic compounds with OH radical [16,17]. Additionally, the calculated *k*_OH_ were applied to the theoretical consideration of persistence of PCDDs.

## 2. Results and Discussion

### 2.1. Reactions with OH Radicals

**Figure 1:**
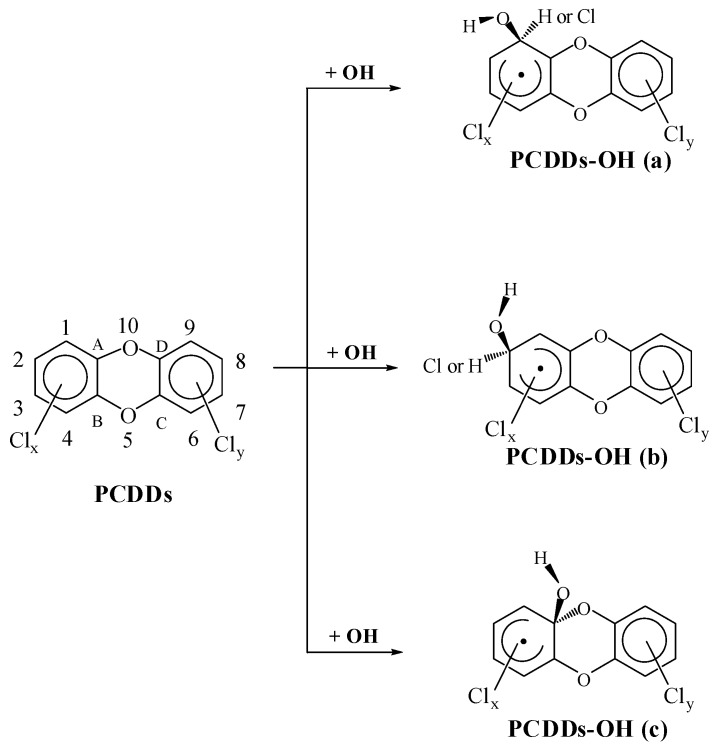
Schematic pathways for OH radical addition of PCDDs.

There are two channels in the reactions of OH radical with PCDDs, *i.e.*, OH radical addition and H atom abstraction. Generally, the H atom abstraction is not dominant for PCDDs and can be negligible [18]. So here we focus on the addition reactions between PCDDs and OH radical. The scheme for OH radical addition pathways are shown in Figure 1. Three different carbon sites, *C*_α_ (1, 4, 6, 9), *C*_β_ (2, 3, 7, 8), and *C*_γ_ (*A*, *B*, *C*, *D*), can be added by OH radical.

### 2.2. Relation between Rate Constants and the Configuration Parameters

The configuration parameters of PCDDs include the numbers and positions of Cl atoms, the energy of the highest occupied molecular orbital (*E*_HOMO_), the energy of the lowest unoccupied molecular orbital (*E*_LUMO_), and the dipole of oxidizing agents (*D*).

The number and the positions of Cl substitution are defined as follows: the number of Cl atoms at positions α (2, 3, 7, 8) and positions β (1, 4, 6, 9) are denoted as *N*_α_ and *N*_β_, the number of Cl atoms at positions 1 and 9 (or 4 and 6) and positions 2 and 8 (or 3 and 7) are symbolized as *N*_1,9_ and *N*_2,8_, and the pair numbers of ortho-, meta- and para- positions are defined as *N*_o_, *N*_m_ and *N*_p_ where both Cl atoms are located on one benzene ring. The total number of Cl atoms is defined as N. After the frequency at the MPWB1K/6-31+G(d,p) level are calculated, the *E*_HOMO_, *E*_LUMO_ and *D* can be obtained.

The GFA (genetic function approximation) calculations are performed, the correlation of rate constants and the configuration parameters can be obtained, and the results are given in the following.

#### 2.2.1. *k*_α_, *k*_β_ and *k*_γ_ with the Configuration Parameters

**Figure 2:**
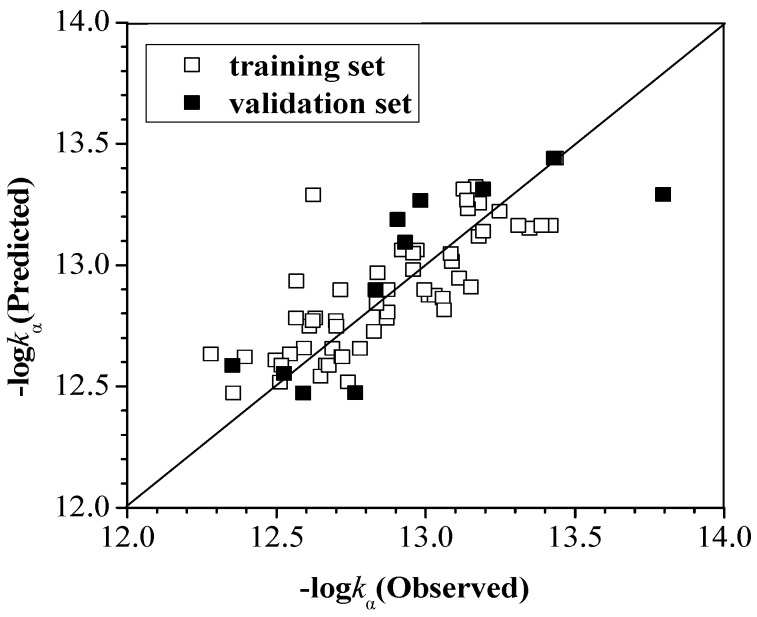
The observed values and predicted values of −log*k*_α_ for the training and validation set.

**Figure 3:**
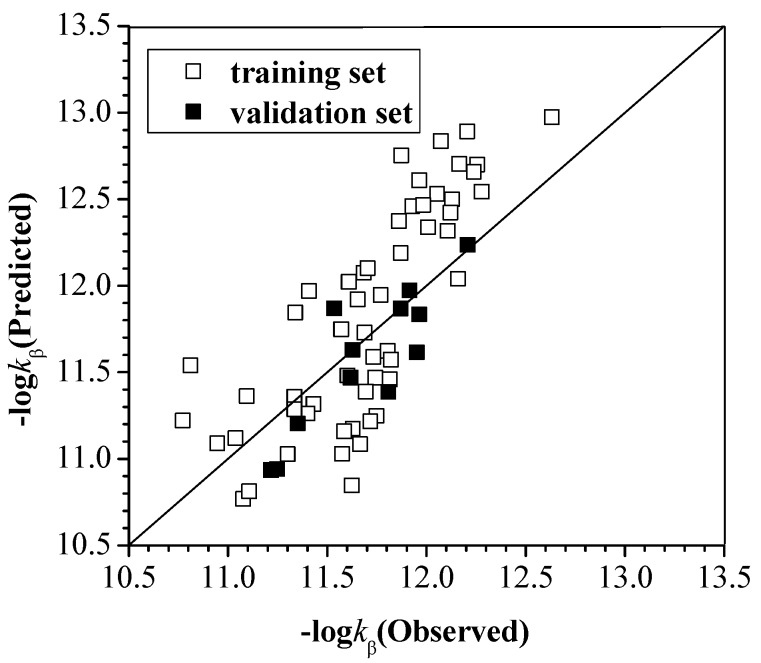
The observed values and predicted values of −log*k*_β_ for the training and validation set.

**Figure 4:**
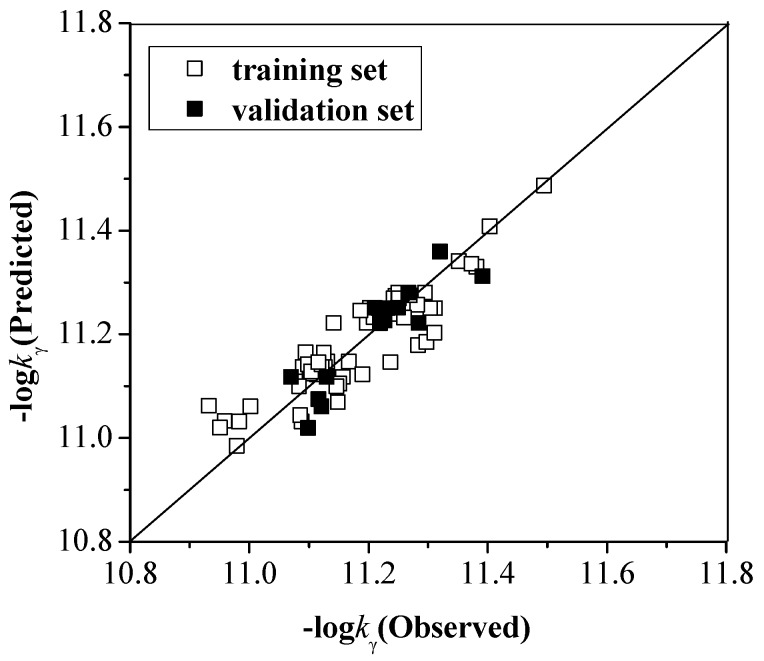
The observed values and predicted values of −log*k*_γ_ for the training and validation set.

*k*_α_, *k*_β_ and *k*_γ_ for all 75 PCDDs with OH radical at 298.15 K are listed in Table 1, Table 2 and Table 3. After performing GFA calculations, we arrive at expressions connecting log*k*_α_, log*k*_β_ and log*k*_γ_ to the configuration parameters which are referred to as model (1), model (2) and model (3), respectively. These equations show how the number and positions of Cl atoms, *E*_HOMO_, *E*_LUMO_ and D influence rate constants. The observed and predicted values are compared in Figure 2, Figure 3 and Figure 4, which can suggest the relativity between the actual values and predicted ones.

**Table 1:** The rate constants (*k*_α_, *k*_β_, *k*_γ_) of MCDDs, DCDDs and Tri-CDDs with parameters.

Compound	*k*_α_	*k*_β_	*k*_γ_	*N*_α_	*N*_β_	*N*_1,9_	*N*_2,8_	*N*_o_	*N*_m_	*N*_p_	*N*	*E*_HOMO_	*E*_LUMO_	Δ*E*_HOMO-LUMO_	*D*
MCDDs															
1	1.35 × 10^−13^	9.15 × 10^−12^	7.40 × 10^−12^	1	0	1	0	0	0	0	1	−0.247	−0.003	0.245	1.63
2	3.18 × 10^−13^	1.69 × 10^−11^	1.12 × 10^−11^	0	1	0	1	0	0	0	1	−0.246	−0.003	0.243	2.07
DCDDs															
1,2	8.66 × 10^−14^	3.98 × 10^−12^	8.21 × 10^−12^	1	1	1	1	1	1	0	2	−0.251	−0.010	0.242	2.86
1,3	4.44 × 10^−13^	5.66 × 10^−12^	7.97 × 10^−12^	1	1	1	0	0	1	0	2	−0.253	−0.011	0.242	2.29
1,4	8.23 × 10^−14^	1.13 × 10^−11^	5.50 × 10^−12^	2	0	1	0	0	0	1	2	−0.255	−0.011	0.244	0.88
1,6	5.25 × 10^−13^	1.54 × 10^−11^	5.62 × 10^−12^	2	0	1	0	0	0	0	2	−0.255	−0.010	0.245	0.00
1,7	2.34 × 10^−13^	8.03 × 10^−12^	6.81 × 10^−12^	1	1	1	0	0	0	0	2	−0.253	−0.011	0.243	1.52
1,8	2.72 × 10^−13^	7.81 × 10^−12^	8.25 × 10^−12^	1	1	1	1	0	0	0	2	−0.253	−0.011	0.242	2.79
1,9	2.85 × 10^−13^	8.39 × 10^−12^	5.57 × 10^−12^	2	0	2	0	0	0	0	2	−0.255	−0.010	0.245	2.99
2,3	3.07 × 10^−13^	4.58 × 10^−12^	7.92 × 10^−12^	0	2	0	1	1	0	0	2	−0.251	−0.009	0.242	3.16
2,7	2.45 × 10^−13^	3.90 × 10^−12^	1.10 × 10^−11^	0	2	0	1	0	0	0	2	−0.252	−0.012	0.241	0.00
2,8	1.99 × 10^−13^	4.63 × 10^−12^	1.05 × 10^−11^	0	2	0	2	0	0	0	2	−0.252	−0.012	0.240	1.79
Tri-CDDs															
1,2,3	2.11 × 10^−13^	2.46 × 10^−12^	7.40 × 10^−12^	1	2	1	1	2	1	0	3	−0.256	−0.015	0.241	3.42
1,2,4	1.04 × 10^−13^	6.06 × 10^−12^	7.66 × 10^−12^	2	1	1	1	1	1	1	3	−0.258	−0.018	0.240	2.40
1,2,6	8.76 × 10^−14^	1.85 × 10^−12^	5.61 × 10^−12^	2	1	1	1	1	0	0	3	−0.259	−0.017	0.242	1.59
1,2,7	9.32 × 10^−14^	2.22 × 10^−12^	7.55 × 10^−12^	1	2	1	1	1	0	0	3	−0.257	−0.017	0.240	1.14
1,2,8	9.77 × 10^−14^	1.81 × 10^−12^	8.14 × 10^−12^	1	2	1	2	1	0	0	3	−0.257	−0.017	0.240	2.89
1,2,9	7.05 × 10^−14^	2.66 × 10^−12^	5.68 × 10^−12^	2	1	2	1	1	0	0	3	−0.258	−0.016	0.242	3.76
1,3,6	4.03 × 10^−13^	4.99 × 10^−12^	7.66 × 10^−12^	2	1	1	0	0	1	0	3	−0.260	−0.018	0.242	1.96
1,3,7	2.16 × 10^−13^	1.57 × 10^−12^	8.17 × 10^−12^	1	2	1	0	0	1	0	3	−0.259	−0.019	0.240	0.48
1,3,8	3.04 × 10^−13^	4.63 × 10^−12^	1.04 × 10^−11^	1	2	1	1	0	1	0	3	−0.259	−0.019	0.240	1.51
1,3,9	1.90 × 10^−13^	2.38 × 10^−12^	5.80 × 10^−12^	2	1	2	0	0	1	0	3	−0.260	−0.018	0.242	2.66
1,4,6	7.72 × 10^−14^	2.60 × 10^−12^	3.95 × 10^−12^	3	0	1	0	0	0	1	3	−0.262	−0.017	0.245	1.48
1,4,7	1.17 × 10^−13^	4.45 × 10^−12^	5.93 × 10^−12^	2	1	1	0	0	0	1	3	−0.260	−0.018	0.242	1.27
2,3,6	2.98 × 10^−13^	2.92 × 10^−12^	5.63 × 10^−12^	1	2	0	1	1	0	0	3	−0.258	−0.016	0.242	3.06
2,3,7	1.82 × 10^−13^	1.04 × 10^−12^	7.47 × 10^−12^	0	3	0	1	1	0	0	3	−0.257	−0.017	0.240	1.58

**Table 2:** The rate constants (*k*_α_, *k*_β_, *k*_γ_) of TCDDs with parameters.

Compound	*k*_α_	*k*_β_	*k*_γ_	*N*_α_	*N*_β_	*N*_1,9_	*N*_2,8_	*N*_o_	*N*_m_	*N*_p_	*N*	*E*_HOMO_	*E*_LUMO_	Δ*E*_HOMO-LUMO_	*D*
TCDDs															
1,2,3,4	6.62 × 10^−14^	2.07 × 10^−12^	7.06 × 10^−12^	2	2	1	1	3	2	1	4	−0.260	−0.022	0.239	3.30
1,2,3,6	1.91 × 10^−13^	1.35 × 10^−12^	5.38 × 10^−12^	2	2	1	1	2	1	0	4	−0.263	−0.022	0.241	2.77
1,2,3,7	2.00 × 10^−13^	1.34 × 10^−12^	7.56 × 10^−12^	1	3	1	1	2	1	0	4	−0.261	−0.023	0.239	1.42
1,2,3,8	2.39 × 10^−13^	7.45 × 10^−13^	7.97 × 10^−12^	1	3	1	2	2	1	0	4	−0.261	−0.023	0.239	2.40
1,2,3,9	1.34 × 10^−13^	1.70 × 10^−12^	5.23 × 10^−12^	2	2	2	1	2	1	0	4	−0.262	−0.022	0.241	3.71
1,2,4,6	4.89 × 10^−14^	1.79 × 10^−12^	4.88 × 10^−12^	3	1	1	1	1	1	1	4	−0.265	−0.024	0.241	2.05
1,2,4,7	6.42 × 10^−14^	1.09 × 10^−12^	7.42 × 10^−12^	2	2	1	1	1	1	1	4	−0.263	−0.025	0.239	0.61
1,2,4,8	7.46 × 10^−14^	2.02 × 10^−12^	7.10 × 10^−12^	2	2	1	2	1	1	1	4	−0.263	−0.025	0.238	1.48
1,2,4,9	3.81 × 10^−14^	2.16 × 10^−12^	4.97 × 10^−12^	3	1	2	1	1	1	1	4	−0.265	−0.024	0.241	2.68
1,2,6,7	6.42 × 10^−14^	1.18 × 10^−12^	6.06 × 10^−12^	2	2	1	1	2	0	0	4	−0.262	−0.023	0.239	0.00
1,2,6,8	1.47 × 10^−13^	2.36 × 10^−12^	8.51 × 10^−12^	2	2	1	2	1	1	0	4	−0.263	−0.024	0.239	1.41
1,2,6,9	5.65 × 10^−14^	1.92 × 10^−12^	4.16 × 10^−12^	3	1	2	1	1	0	1	4	−0.265	−0.023	0.241	2.17
1,2,7,8	1.45 × 10^−13^	8.84 × 10^−13^	6.35 × 10^−12^	1	3	1	2	2	0	0	4	−0.261	−0.023	0.239	2.28
1,2,7,9	1.93 × 10^−13^	1.51 × 10^−12^	8.04 × 10^−12^	2	2	2	1	1	1	0	4	−0.263	−0.024	0.240	2.49
1,2,8,9	6.79 × 10^−14^	1.53 × 10^−12^	7.22 × 10^−12^	2	2	2	2	2	0	0	4	−0.261	−0.023	0.239	3.88
1,3,6,8	4.41 × 10^−13^	2.69 × 10^−12^	9.96 × 10^−12^	2	2	1	1	0	2	0	4	−0.265	−0.025	0.240	0.00
1,3,6,9	2.71 × 10^−13^	2.35 × 10^−12^	5.68 × 10^−12^	3	1	2	0	0	1	1	4	−0.267	−0.025	0.242	1.39
1,3,7,8	2.25 × 10^−13^	1.38 × 10^−12^	6.97 × 10^−12^	1	3	1	1	1	1	0	4	−0.263	−0.024	0.239	1.08
1,3,7,9	2.57 × 10^−13^	2.41 × 10^−12^	7.57 × 10^−12^	2	2	2	0	0	2	0	4	−0.265	−0.025	0.240	1.08
1,4,6,9	3.93 × 10^−16^	3.70 × 10^−12^	3.20 × 10^−12^	4	0	2	0	0	0	2	4	−0.269	−0.024	0.244	0.00
1,4,7,8	1.24 × 10^−13^	1.35 × 10^−12^	4.06 × 10^−12^	2	2	1	1	1	0	1	4	−0.264	−0.024	0.241	2.21
2,3,7,8	1.72 × 10^−13^	1.35 × 10^−15^	5.20 × 10^−12^	0	4	0	2	2	0	0	4	−0.261	−0.022	0.239	0.00

**Table 3:** The rate constants (*k*_α_, *k*_β_, *k*_γ_) of Penta-CDDs, Hexa-CDDs, Hepta-CDDs and OCDD with parameters.

Compound	*k*_α_	*k*_β_	*k*_γ_	*N*_α_	*N*_β_	*N*_1,9_	*N*_2,8_	*N*_o_	*N*_m_	*N*_p_	*N*	*E*_HOMO_	*E*_LUMO_	Δ*E*_HOMO-LUMO_	*D*
Penta-CDDs															
1,2,3,4,6	4.48 × 10^−14^	9.83 × 10^−13^	5.07 × 10^−12^	3	2	1	1	3	2	1	5	−0.267	−0.028	0.240	3.14
1,2,3,4,7	4.09 × 10^−14^	6.84 × 10^−13^	7.14 × 10^−12^	2	3	1	1	3	2	1	5	−0.265	−0.028	0.237	1.70
1,2,3,6,7	1.34 × 10^−13^	2.34 × 10^−13^	6.10 × 10^−12^	2	3	1	1	3	1	0	5	−0.266	−0.028	0.238	1.51
1,2,3,6,8	2.05 × 10^−13^	1.09 × 10^−12^	7.71 × 10^−12^	2	3	1	2	2	2	0	5	−0.267	−0.029	0.238	1.12
1,2,3,6,9	1.10 × 10^−13^	6.93 × 10^−13^	4.24 × 10^−12^	3	2	2	1	2	1	1	5	−0.269	−0.028	0.240	2.49
1,2,3,7,8	1.49 × 10^−13^	1.24 × 10^−15^	5.21 × 10^−12^	1	4	1	2	3	1	0	5	−0.265	−0.028	0.237	1.06
1,2,3,7,9	1.66 × 10^−13^	7.56 × 10^−13^	6.46 × 10^−12^	2	3	2	1	2	2	0	5	−0.267	−0.029	0.238	1.84
1,2,3,8,9	1.01 × 10^−13^	5.26 × 10^−13^	6.51 × 10^−12^	2	3	2	2	3	1	0	5	−0.266	−0.028	0.238	3.18
1,2,4,6,7	3.66 × 10^−14^	1.98 × 10^−12^	5.72 × 10^−12^	3	2	1	1	2	1	1	5	−0.268	−0.030	0.239	1.39
1,2,4,6,8	2.38 × 10^−13^	2.50 × 10^−12^	1.17 × 10^−11^	3	2	1	2	1	2	1	5	−0.267	−0.029	0.238	1.12
1,2,4,6,9	7.33 × 10^−16^	1.56 × 10^−12^	4.79 × 10^−12^	4	1	2	1	1	1	2	5	−0.271	−0.030	0.241	1.51
1,2,4,7,8	7.27 × 10^−14^	5.78 × 10^−13^	4.89 × 10^−12^	2	3	1	2	2	1	1	5	−0.267	−0.030	0.238	0.93
1,2,4,7,9	8.16 × 10^−14^	2.05 × 10^−12^	7.50 × 10^−12^	3	2	2	1	1	2	1	5	−0.270	−0.031	0.239	1.02
1,2,4,8,9	3.71 × 10^−14^	1.12 × 10^−12^	6.03 × 10^−12^	3	2	2	2	2	1	1	5	−0.268	−0.030	0.238	2.41
Hexa-CDDs															
1,2,3,4,6,7	1.60 × 10^−14^	2.54 × 10^−13^	6.16 × 10^−12^	3	3	1	1	4	2	1	6	−0.270	−0.033	0.237	2.26
1,2,3,4,6,8	1.10 × 10^−13^	8.46 × 10^−13^	7.87 × 10^−12^	3	3	1	2	3	3	1	6	−0.271	−0.034	0.237	1.19
1,2,3,4,6,9	7.74 × 10^−16^	7.82 × 10^−13^	4.45 × 10^−12^	4	2	2	1	3	2	2	6	−0.273	−0.034	0.239	2.36
1,2,3,4,7,8	6.63 × 10^−14^	9.51 × 10^−16^	5.04 × 10^−12^	2	4	1	2	4	2	1	6	−0.269	−0.033	0.236	0.19
1,2,3,6,7,8	2.56 × 10^−13^	1.15 × 10^−15^	6.27 × 10^−12^	2	4	1	2	4	2	0	6	−0.270	−0.033	0.237	0.00
1,2,3,6,7,9	1.07 × 10^−13^	6.23 × 10^−13^	6.05 × 10^−12^	3	3	2	1	3	2	1	6	−0.272	−0.034	0.237	0.97
1,2,3,6,8,9	1.20 × 10^−13^	5.56 × 10^−13^	6.32 × 10^−12^	3	3	2	2	3	2	1	6	−0.272	−0.034	0.237	1.71
1,2,3,7,8,9	1.46 × 10^−13^	1.13 × 10^−15^	6.19 × 10^−12^	2	4	2	2	4	2	0	6	−0.269	−0.033	0.237	2.01
1,2,4,6,7,9	6.30 × 10^−16^	6.19 × 10^−13^	6.07 × 10^−12^	4	2	2	1	2	2	2	6	−0.274	−0.036	0.238	0.00
1,2,4,6,8,9	6.55 × 10^−16^	1.22 × 10^−12^	5.86 × 10^−12^	4	2	2	2	2	2	2	6	−0.274	−0.036	0.238	1.02
Hepta-CDDs															
1,2,3,4,6,7,8	7.21 × 10^−14^	9.28 × 10^−16^	5.71 × 10^−12^	3	4	1	2	5	3	1	7	−0.273	−0.038	0.236	0.95
1,2,3,4,6,7,9	6.81 × 10^−16^	4.15 × 10^−13^	5.41 × 10^−12^	4	3	2	1	4	3	2	7	−0.276	−0.039	0.237	1.06
OCDD															
1,2,3,4,6,7,8,9	7.95 × 10^−16^	7.51 × 10^−16^	5.98 × 10^−12^	4	4	2	2	6	4	2	8	−0.277	−0.043	0.235	0.00

(1) log*k*_α_ = −12.50 − 0.064 × *N*_α_ − 0.880 × *N*_p_, *R*^2^ = 0.761, *R*^2^(*CV*) = 0.715, *Q*^2^*ext* = 0.716.

(2) log*k*_β_ = 42.950 − 0.660 × *N*_β_ − 0.467 × *N*_o_ − 222.406 × Δ*E*_HOMO-LUMO_ + 0.258 × *D*, *R*^2^ = 0.835, *R*^2^(*CV*) = 0.821, *Q*^2^*ext* = 0.733.

(3) log*k*_γ_ = 5.179 − 0.062 × *N*_o_ + 0.080 × *N*_m_ + 18.188 × *E*_HOMO_ − 48.247 × Δ*E*_HOMO-LUMO_, *R*^2^ = 0.878, *R*^2^(*CV*) = 0.866, *Q*^2^*ext* = 0.743.

From model (1), it can be shown that the correlation coefficient *R*^2^ and the cross validation *R*^2^(*CV*) are not ideal, but still show some useful information. The coefficient of *N*_α_ is 0.064, while the coefficient of *N*_p_ is 0.880. The effect of relative positions of these Cl atoms is more important than the number of these Cl atoms. As described in the algorithm and the methodology section, we have separately performed steps 1 to 4 and calculated *S_new_*_(*k*)_ value for training and test set compounds. All the PCDDs in the training set are not *X*-outlier, and all the PCDDs in the test set are within applicability domain.

As shown by the *R*^2^ and *R*^2^(*CV*) in model (2) and model (3), the *k*_β_ and *k*_γ_ are well correlated to the variables. The relativity between the actual values and predicted values is excellent as shown in the Figure 3 and Figure 4. The *k*_β_ is positively correlated with *N*_β_, *N*_o_ and Δ*E*_HOMO-LUMO_. The effect of relative positions and the number of these Cl atoms is in the order of *N*_β_ > *N*_o_. The *k*_γ_ is positively correlated with *E*_HOMO_ and negatively correlated with Δ*E*_HOMO-LUMO_. But the *k*_γ_ is not related to the number of these Cl atoms (*N*_α_, *N*_β_ and *N*) and it has little to do with the number of relative position for these Cl atoms (*N*_o_, *N*_m_, *N*_p_, *N*_1,9_ and *N*_2,8_) because its coefficient is less than those of other variables. The applicability domain of the model (2) and model (3) were checked; the training set compound OCDD was considered as an outlier, and the *S_new_* value of this compound was 3.02.

#### 2.2.2. The Total Rate Constant *k*_OH_ with the Configuration Parameters

**Figure 5:**
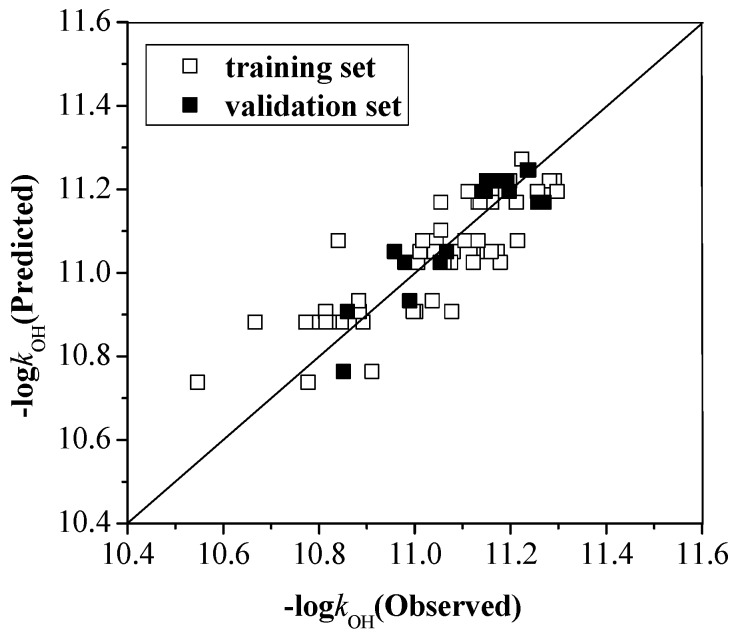
The observed values and predicted values of –log*k*_OH_ for the training and validation set.

The total rate constant of the addition reaction between PCDDs and the OH radical is *k*_OH_ *= k*_α_ + *k*_β_ + *k*_γ_. Using GFA calculations, the correlation of *k*_OH_ with the configuration parameters is given in model (4), where the *R*^2^ is 0.865 and the *R*^2^(*CV*) is 0.852. As revealed in Figure 5, the actual values and predicted values seem pretty relevant.

(4) log*k*_OH_ = −10.594 + 0.118 × *N*_o_ − 0.144 × *N*, *R*^2^ = 0.865, *R*^2^(*CV*) = 0.852, *Q*^2^*ext* = 0.806.

Several important conclusions can be drawn from the model (4). First, the main influence factor of *k*_OH_ is the *N*, and *k*_OH_ is positively correlated with the *N*_o_. Second, the number of Cl atoms is more effective than the number of relative position for these Cl atoms because the coefficients of *N* is greater than that of *N*_o_. Finally, the Cl atom located at the ortho-position exerts more effect on the *k*_OH_. The applicability domain of the model (4) have been checked, the training set compound OCDD is considered as an outlier, and the *S_new_* value of this compound is 3.02.

## 3. Computational Methods

### 3.1. Geometry Optimization

Using the Gaussian 03 programs [19], high-level *ab initio* molecular orbital calculations were carried out for the 75 PCDDs and OH radical. The geometrical parameters of the 75 PCDDs, transition states (TS), and OH-adducts are optimized at the MPWB1K/6-31+G(d,p) level. The MPWB1K method is a hybrid density functional theory model with excellent performance for thermochemistry and thermochemical kinetics [20,21]. This computational approach has been successfully used in TCDD and TCDF [22,23]. All open-shell species were treated with an unrestricted approach, while closed-shell species were described with a restricted method. Structure optimizations were performed in Cartesian coordinates with an energy convergence criterion of 10^−6^ Hartree. Vibrational frequencies, calculated at the same level, were scaled by a standard scaling factor of 0.934 to remove systematic errors [24]. Each transition state was verified to connect the designated reactants and products by performing an intrinsic reaction coordinate (IRC) analysis [25].

### 3.2. Kinetic Calculation

Based on the quantum chemical information, rate constants are computed using the Rice–Ramsperger–Kassel–Marcus (RRKM) theory [26]. The microcannonical rate constants were calculated using RRKM:

(5) $$k_{i}\left(E\right)={\text{α}}_{i}\;\kappa_{i}\sqrt{\frac{I_{i}^{\neq }}{I_{j}^{IM}}\;}\frac{N\left(E-E_{i}^{\neq }\right)}{h{\text{ρ}}_{j}\left(E\right)},\;i\;=\;1,2,3\ldots ;\;\;j\;=\;1,2,3\ldots$$

where α*_i_* is the statistical factor for the path degeneracy, κ*_i_* is the tunneling factor, $I_{i}^{\neq }$ and $I_{J}^{IM}$ are the moments of inertia (*I*a*I*b*I*c) of transition sate *i* and intermediate *j*, and *N*(*E* − $E_{i}^{\neq }$) is the number of states with energy above the barrier height $E_{i}^{\neq }$ for *i*. The Beyer–Swinehart algorithm was employed to calculate the density and number of states.

### 3.3. Quantitative Structure–Activity Relationship

The genetic function approximation (GFA) [27] in the Materials studio package is used to analyze the relationship between the OH radical addition rate constants and configuration parameters [28]. In order to improve the correlation of mathematic model, the exceptional values in test data are eliminated after outlier analysis.

In absence of any new data, for all the datasets, we picked one compound out of every five to constitute validation sets and the rest constituted the training set. The list of chemicals used for developing models along with the division of training and validation sets is given in Tables S1 and S2.

The external predictive ability of the models may be shown by using different metric values [29]. In this study, it was tested through test set and evaluated by cross-validation coefficient *Q*^2^*ext* between predicted and observed values. *Q*^2^*ext* = 1 − PRESS/SD, where PRESS is the sum of squared differences between the experimental values and the predicted value for each molecule in the test set, and SD is the sum of squared deviations between the experimental values for each molecule in the test set and the mean experimental value of the training set [30].

The reliability of any QSAR model depends on the confident predictions of these new compounds based on the applicability domain of the model. The algorithm and methodology for the proposed approach are discussed below [31]:

(1) First, all the descriptors appearing in the developed model (for training and test set compounds) are standardized using the following formula:

(6) $$S_{ki}=\frac{\left|X_{ki}-\bar{X_{i}}\right|}{{\text{σ}}_{X_{i}}}$$

where *k* is the number of compounds, *i* is the number of descriptors, *S_ki_* is standardized descriptor *i* for compound *k* (from the training or test set), *X_ki_* is the original descriptor *i* for compound *k* (from the training or test set), $\bar{X_{i}}$ is the mean value of the descriptor *X_i_* for the training set compounds only, and ${\text{σ}}_{X_{i}}$ is standard deviation of the descriptor *X_i_* for the training set compounds only.

(2) Thereafter, one needs to compute the maximum *S_i_*_(*k*)_ value ([*S_i_*]*_max_*_(*k*)_) for the compound *k*. If [*S_i_*]*_max_*_(*k*)_ is lower than or equal to 3, then that compound is not an *X*-outlier (if in the training set) or is within the applicability domain (if in the test set).

(3) If [*S_i_*]*_max_*_(*k*)_ is above 3, then one should compute [*S_i_*]*_min_*_(*k*)_. If [*S_i_*]*_min_*_(*k*)_ > 3, then the compound is an *X*-outlier (if in the training set) or is not within applicability domain (if in the test set).

(4) If [*S_i_*]*_max_*_(*k*)_ > 3and [*S_i_*]*_min_*_(*k*)_ < 3, then one should compute *S_new_*_(*k*)_ from the following equation:

(7) $$S_{new(k)}=\bar{S_{k}}\;+\;1.28\;\times \;\sigma_{S_{k}}$$

where *S_new_*_(*k*)_ is the *S_new_* value for the compound *k*, $\bar{S_{k}}$ is the mean of *S_i_*_(*k*)_ value for the compound *k*, and ${\text{σ}}_{S_{k}}$ is the standard deviation of *S_i_*_(*k*)_ values of the compound *k*.

If the calculated *S_new_*_(*k*)_ value is lower than or equal to 3, then that compound is not an *X*-outlier (if in the training set) or is within applicability domain (if in the test set).

## 4. Conclusions

The atmospheric chemical mechanism of all 75 PCDDs initiated by the OH radical was investigated. Rate constants of the elementary reactions at 298.15 K were calculated. In addition, the rate constants (*k*_α_, *k*_β_, *k*_γ_ and *k*_OH_) were further quantified by the study on QSAR. According to the QSAR models, some valuable conclusions can be drawn:

(1) The *k*_α_ is negatively correlated with the *N*_α_ and the *k*_β_ is negatively correlated with the *N*_β_, while the *k*_γ_ is not related to the number of these Cl atoms (*N*_α_, *N*_β_ and *N*).

(2) The number of Cl atoms is the main influence factor and is positively correlated with the *k*_OH_. For the *k*_OH_, the number of Cl atoms is more effective than the relative position for these Cl atoms. The *k*_OH_ decreases with the increase of the substitute number of Cl atoms.

## Supplementary Materials

Supplementary materials can be found at http://www.mdpi.com/1422-0067/16/08/18812/s1.
