# Supplementary material for: Quantitative Structure-Activity Relationships Study on the Rate Constants of Polychlorinated Dibenzo-p-Dioxins with OH Radical
Source: Int J Mol Sci. 2015 Aug 12;16(8):18812–24. doi: 10.3390/ijms160818812 (PMC4581273; doi:10.3390/ijms160818812)
Supplement: Supplementary file 1 [file ijms-16-18812-s001.pdf]

Supplementary Information

Table S1. The training sets of the rate constants ($k_α$, $k_β$, $k_γ$) and parameters.

Compound	$k_α$	$k_β$	$k_γ$	$N_α$	$N_β$	$N_{1,9}$	$N_{2,8}$	N_o	N_m	N_p	N	E_{HOMO}	E_{LUMO}	$ΔE_{HOMO-LUMO}$	D
1,2,3,6,8,9	1.20E-13	5.56E-13	6.32E-12	3	3	2	2	3	2	1	6	−0.272	−0.034	0.237	0.53
2,7	2.45E-13	3.90E-12	1.10E-11	0	2	0	1	0	0	0	2	−0.252	−0.012	0.241	0.15
1,4,6,9	3.93E-16	3.70E-12	3.20E-12	4	0	2	0	0	0	2	4	−0.269	−0.024	0.244	0.23
1,2,3,6,7,8	2.56E-13	1.15E-15	6.27E-12	2	4	1	2	4	2	0	6	−0.27	−0.033	0.237	0.55
1,2,8	9.77E-14	1.81E-12	8.14E-12	1	2	1	2	1	0	0	3	−0.257	−0.017	0.24	0.11
1,2,3,6,7,9	1.07E-13	6.23E-13	6.05E-12	3	3	2	1	3	2	1	6	−0.272	−0.034	0.237	0.55
1,2,3,4,6	4.48E-14	9.83E-13	5.07E-12	3	2	1	1	3	2	1	5	−0.267	−0.028	0.24	0.56
1	1.35E-13	9.15E-12	7.40E-12	1	0	1	0	0	0	0	1	−0.247	−0.003	0.245	−0.03
1,2,3,6,8	2.05E-13	1.09E-12	7.71E-12	2	3	1	2	2	2	0	5	−0.267	−0.029	0.238	0.41
1,2,4,6	4.89E-14	1.79E-12	4.88E-12	3	1	1	1	1	1	1	4	−0.265	−0.024	0.241	0.32
2	3.18E-13	1.69E-11	1.12E-11	0	1	0	1	0	0	0	1	−0.246	−0.003	0.243	0.08
1,2,4,9	3.81E-14	2.16E-12	4.97E-12	3	1	2	1	1	1	1	4	−0.265	−0.024	0.241	0.25
1,3,7	2.16E-13	1.57E-12	8.17E-12	1	2	1	0	0	1	0	3	−0.259	−0.019	0.24	0.20
1,9	2.85E-13	8.39E-12	5.57E-12	2	0	2	0	0	0	0	2	−0.255	−0.01	0.245	−0.09
1,3,6,9	2.71E-13	2.35E-12	5.68E-12	3	1	2	0	0	1	1	4	−0.267	−0.025	0.242	0.23
1,2,8,9	6.79E-14	1.53E-12	7.22E-12	2	2	2	2	2	0	0	4	−0.261	−0.023	0.239	0.09
1,2,3,4,6,7,8,9	7.95E-16	7.51E-16	5.98E-12	4	4	2	2	6	4	2	8	−0.277	−0.043	0.235	1.06
1,2,7	9.32E-14	2.22E-12	7.55E-12	1	2	1	1	1	0	0	3	−0.257	−0.017	0.24	0.13
1,2,4,8	7.46E-14	2.02E-12	7.10E-12	2	2	1	2	1	1	1	4	−0.263	−0.025	0.238	0.36
1,2,7,9	1.93E-13	1.51E-12	8.04E-12	2	2	2	1	1	1	0	4	−0.263	−0.024	0.24	0.16
1,4,6	7.72E-14	2.60E-12	3.95E-12	3	0	1	0	0	0	1	3	−0.262	−0.017	0.245	0.14
2,3	3.07E-13	4.58E-12	7.92E-12	0	2	0	1	1	0	0	2	−0.251	−0.009	0.242	0.18
1,2,3,7,8	1.49E-13	1.24E-15	5.21E-12	1	4	1	2	3	1	0	5	−0.265	−0.028	0.237	0.41
1,2,3,6,7	1.34E-13	2.34E-13	6.10E-12	2	3	1	1	3	1	0	5	−0.266	−0.028	0.238	0.37
1,3,7,8	2.25E-13	1.38E-12	6.97E-12	1	3	1	1	1	1	0	4	−0.263	−0.024	0.239	0.29
1,2,3	2.11E-13	2.46E-12	7.40E-12	1	2	1	1	2	1	0	3	−0.256	−0.015	0.241	0.25
1,7	2.34E-13	8.03E-12	6.81E-12	1	1	1	0	0	0	0	2	−0.253	−0.011	0.243	0.04
1,2,3,8	2.39E-13	7.45E-13	7.97E-12	1	3	1	2	2	1	0	4	−0.261	−0.023	0.239	0.30
1,8	2.72E-13	7.81E-12	8.25E-12	1	1	1	1	0	0	0	2	−0.253	−0.011	0.242	0.02
1,2,3,9	1.34E-13	1.70E-12	5.23E-12	2	2	2	1	2	1	0	4	−0.262	−0.022	0.241	0.19

Table S1. *Cont.*

Compound	$k_α$	$k_β$	$k_γ$	$N_α$	$N_β$	$N_{1,9}$	$N_{2,8}$	N_o	N_m	N_p	N	E_{HOMO}	E_{LUMO}	$ΔE_{HOMO-LUMO}$	D
1,2,6,9	5.65E-14	1.92E-12	4.16E-12	3	1	2	1	1	0	1	4	−0.265	−0.023	0.241	0.16
1,2,3,4	6.62E-14	2.07E-12	7.06E-12	2	2	1	1	3	2	1	4	−0.26	−0.022	0.239	0.54
1,2,3,4,7	4.09E-14	6.84E-13	7.14E-12	2	3	1	1	3	2	1	5	−0.265	−0.028	0.237	0.61
1,2,3,4,7,8	6.63E-14	9.51E-16	5.04E-12	2	4	1	2	4	2	1	6	−0.269	−0.033	0.236	0.70
1,2,7,8	1.45E-13	8.84E-13	6.35E-12	1	3	1	2	2	0	0	4	−0.261	−0.023	0.239	0.21
1,2,9	7.05E-14	2.66E-12	5.68E-12	2	1	2	1	1	0	0	3	−0.258	−0.016	0.242	−0.0008
1,2,6,7	6.42E-14	1.18E-12	6.06E-12	2	2	1	1	2	0	0	4	−0.262	−0.023	0.239	0.18
1,2,3,6	1.91E-13	1.35E-12	5.38E-12	2	2	1	1	2	1	0	4	−0.263	−0.022	0.241	0.27
2,3,7	1.82E-13	1.04E-12	7.47E-12	0	3	0	1	1	0	0	3	−0.257	−0.017	0.24	0.25
1,2,4,6,7	3.66E-14	1.98E-12	5.72E-12	3	2	1	1	2	1	1	5	−0.268	−0.03	0.239	0.43
1,2,3,4,6,8	1.10E-13	8.46E-13	7.87E-12	3	3	1	2	3	3	1	6	−0.271	−0.034	0.237	0.70
1,2,4,6,8	2.38E-13	2.50E-12	1.17E-11	3	2	1	2	1	2	1	5	−0.267	−0.029	0.238	0.46
1,6	5.25E-13	1.54E-11	5.62E-12	2	0	1	0	0	0	0	2	−0.255	−0.01	0.245	−0.02
1,2,3,4,6,7,8	7.21E-14	9.28E-16	5.71E-12	3	4	1	2	5	3	1	7	−0.273	−0.038	0.236	0.84
1,2,3,7,9	1.66E-13	7.56E-13	6.46E-12	2	3	2	1	2	2	0	5	−0.267	−0.029	0.238	0.36
1,3,9	1.90E-13	2.38E-12	5.80E-12	2	1	2	0	0	1	0	3	−0.26	−0.018	0.242	0.07
1,2,3,7	2.00E-13	1.34E-12	7.56E-12	1	3	1	1	2	1	0	4	−0.261	−0.023	0.239	0.32
1,2,3,7,8,9	1.46E-13	1.13E-15	6.19E-12	2	4	2	2	4	2	0	6	−0.269	−0.033	0.237	0.48
1,2,3,4,6,9	7.74E-16	7.82E-13	4.45E-12	4	2	2	1	3	2	2	6	−0.273	−0.034	0.239	0.64
1,2,4,7,9	8.16E-14	2.05E-12	7.50E-12	3	2	2	1	1	2	1	5	−0.27	−0.031	0.239	0.41
1,3,6	4.03E-13	4.99E-12	7.66E-12	2	1	1	0	0	1	0	3	−0.26	−0.018	0.242	0.14
1,4	8.23E-14	1.13E-11	5.50E-12	2	0	1	0	0	0	1	2	−0.255	−0.011	0.244	0.130
1,2,6	8.76E-14	1.85E-12	5.61E-12	2	1	1	1	1	0	0	3	−0.259	−0.017	0.242	0.07
1,2	8.66E-14	3.98E-12	8.21E-12	1	1	1	1	1	1	0	2	−0.251	−0.01	0.242	0.15
1,3,8	3.04E-13	4.63E-12	1.04E-11	1	2	1	1	0	1	0	3	−0.259	−0.019	0.24	0.18
1,2,3,8,9	1.01E-13	5.26E-13	6.51E-12	2	3	2	2	3	1	0	5	−0.266	−0.028	0.238	0.28
2,8	1.99E-13	4.63E-12	1.05E-11	0	2	0	2	0	0	0	2	−0.252	−0.012	0.24	0.13
1,2,4,7,8	7.27E-14	5.78E-13	4.89E-12	2	3	1	2	2	1	1	5	−0.267	−0.03	0.238	0.46
1,2,3,6,9	1.10E-13	6.93E-13	4.24E-12	3	2	2	1	2	1	1	5	−0.269	−0.028	0.24	0.36
1,3,6,8	4.41E-13	2.69E-12	9.96E-12	2	2	1	1	0	2	0	4	−0.265	−0.025	0.24	0.28

Table S2. The validation sets of the rate constants ($k_α$, $k_β$, $k_γ$) and parameters.

Compound	$k_α$	$k_β$	$k_γ$	$N_α$	$N_β$	$N_{1,9}$	$N_{2,8}$	N_o	N_m	N_p	N	E_{HOMO}	E_{LUMO}	$ΔE_{HOMO-LUMO}$	D
1,2,4	1.04E-13	6.06E-12	7.66E-12	2	1	1	1	1	1	1	3	−0.258	−0.018	0.24	2.4
1,2,4,6,8,9	6.55E-16	1.22E-12	5.86E-12	4	2	2	2	2	2	2	6	−0.274	−0.036	0.238	1.02
1,2,4,6,9	7.33E-16	1.56E-12	4.79E-12	4	1	2	1	1	1	2	5	−0.271	−0.03	0.241	1.51
2,3,7,8	1.72E-13	1.35E-15	5.20E-12	0	4	0	2	2	0	0	4	−0.261	−0.022	0.239	0
1,2,6,8	1.47E-13	2.36E-12	8.51E-12	2	2	1	2	1	1	0	4	−0.263	−0.024	0.239	1.41
2,3,6	2.98E-13	2.92E-12	5.63E-12	1	2	0	1	1	0	0	3	−0.258	−0.016	0.242	3.06
1,2,3,4,6,7	1.60E-14	2.54E-13	6.16E-12	3	3	1	1	4	2	1	6	−0.27	−0.033	0.237	2.26
1,2,4,6,7,9	6.30E-16	6.19E-13	6.07E-12	4	2	2	1	2	2	2	6	−0.274	−0.036	0.238	0
1,3	4.44E-13	5.66E-12	7.97E-12	1	1	1	0	0	1	0	2	−0.253	−0.011	0.242	2.29
1,2,3,4,6,7,9	6.81E-16	4.15E-13	5.41E-12	4	3	2	1	4	3	2	7	−0.276	−0.039	0.237	1.06
1,2,4,7	6.42E-14	1.09E-12	7.42E-12	2	2	1	1	1	1	1	4	−0.263	−0.025	0.239	0.61
1,4,7,8	1.24E-13	1.35E-12	4.06E-12	2	2	1	1	1	0	1	4	−0.264	−0.024	0.241	2.21
1,3,7,9	2.57E-13	2.41E-12	7.57E-12	2	2	2	0	0	2	0	4	−0.265	−0.025	0.24	1.08
1,4,7	1.17E-13	4.45E-12	5.93E-12	2	1	1	0	0	0	1	3	−0.26	−0.018	0.242	1.27
1,2,4,8,9	3.71E-14	1.12E-12	6.03E-12	3	2	2	2	2	1	1	5	−0.268	−0.03	0.238	2.41
